# Supplementary material for: A modular effector with a DNase domain and a marker for T6SS substrates
Source: Nat Commun. 2019 Aug 9;10:3595. doi: 10.1038/s41467-019-11546-6 (PMC6688995; doi:10.1038/s41467-019-11546-6)
Supplement: Supplementary file 1 — Supplementary Information [file 41467_2019_11546_MOESM1_ESM.pdf]

Supplementary Information

A modular effector with a DNase domain and a marker for type VI secretion system substrates

Jana et al.

Supplementary Table 1. *Vibrio parahaemolyticus* isolates containing the VgrG1b module.

V. parahaemolyticus isolate	NCBI reference sequence	BioSample	Isolation source	Country	Start position	End position
MVP1	NZ_MQMQ01000118.1	SAMN06061934	Litopenaeus vannamei	Malaysia: Negeri Sembilan	4057	8779
MVP2	NZ_MSBY01000057.1	SAMN06066407	aquaculture pond	Malaysia: Negeri Sembilan	5309	587
MVP6	NZ_MSCA01000128.1	SAMN06066428	aquaculture pond	Malaysia: Negeri Sembilan	5309	587
13-028/A3	NZ_JOKE01000084.1	SAMN02866418	Litopenaeus vannamei	Vietnam	13964	18420
12-009A/1335	NZ_MYFF01000438.1	SAMN06546662	Penaeus vannamei	Vietnam	13594	18316
12-297/B	NZ_MYFG01000470.1	SAMN06546663	Penaeus vannamei	Vietnam	22692	17970
PSU5579	NZ_PEBT01000038.1	SAMN07818924	Litopenaeus vannamei	Thailand: southern	5259	537
20130629002S01	NZ_CP020035.1	SAMN06310878	Litopenaeus vannamei	China	760352	765074
VIP4-0447	NZ_AXNS01000058.1	SAMN02471137	oyster	Hong Kong	31804	27082
VP766	NZ_AOOW01000104.1	SAMN02204305	plankton sample	USA: Washington	7475	2732
901128	NZ_AOPG01000002.1	SAMN02204315	stool sample	USA: Washington	9034	4296
S048	NZ_AWLN01000185.1	SAMN02338909	seafood	USA	2646	7368
S046	NZ_AWLP01000239.1	SAMN02338907	environment	Spain	2647	7369

Supplementary Figures

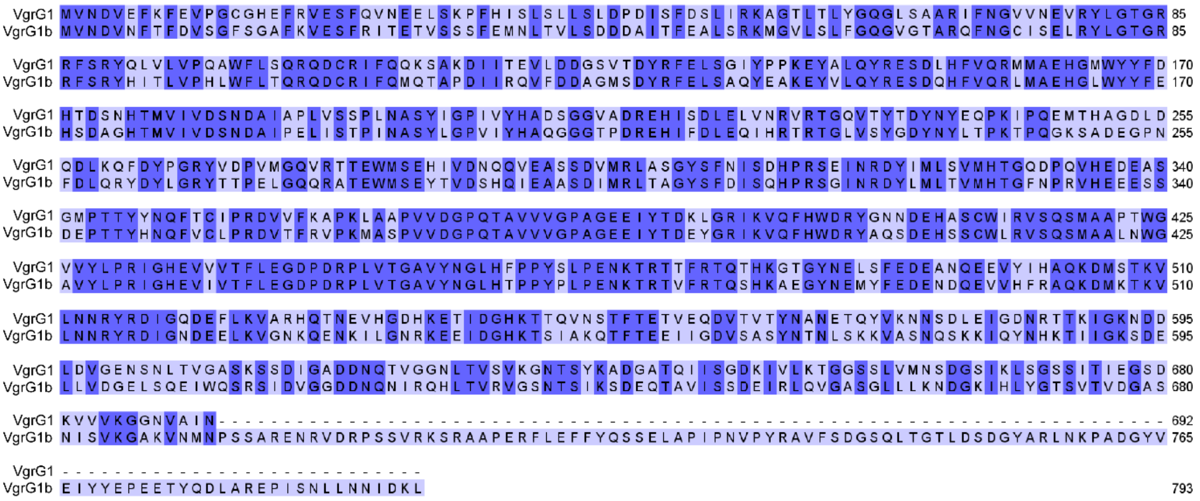

Supplementary Figure 1. VgrG1b is a homolog of VgrG1. Sequence alignment of *V. parahaemolyticus* 12-297/B VgrG1 (B5C30_15295) and VgrG1b (B5C30_14470). Amino acid positions are shown on the right.

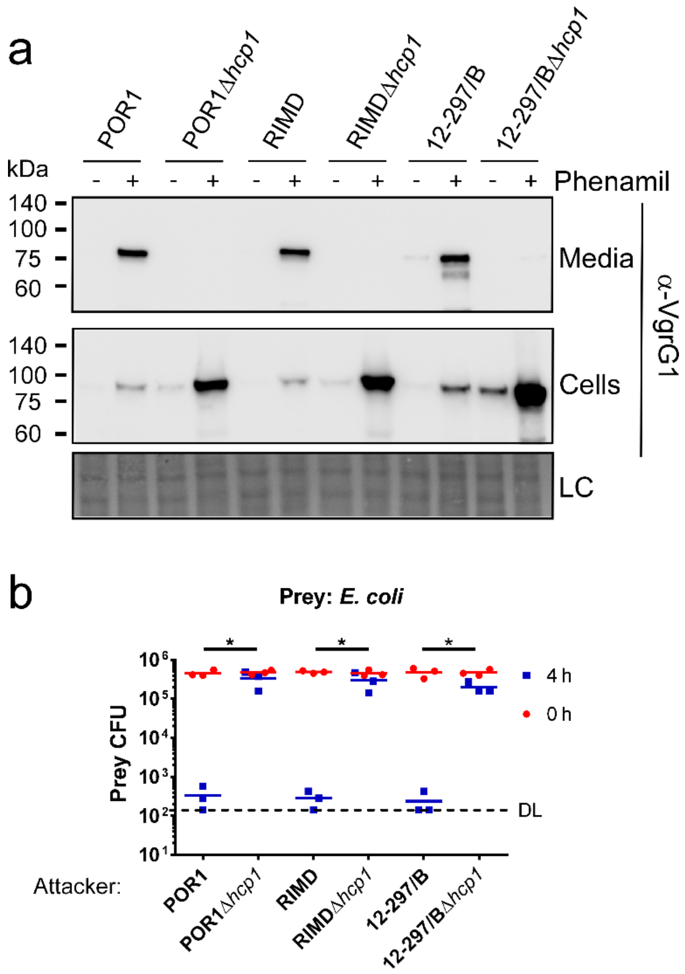

Supplementary Figure 2. *V. parahaemolyticus* 12-297/B T6SS1 mediates antibacterial activity under warm marine-like conditions. a, Expression (cells) and secretion (media) of VgrG1 were detected by immunoblotting using specific antibodies against VgrG1. Loading control (LC), visualized as trihalo compounds' fluorescence of the immunoblot membrane, is shown for total protein lysates. Phenamil (20 µM) was added to induce surface sensing in suspension. **b,** Viability counts of *E. coli* prey before (0 h) and after (4 h) co-incubation with the indicated *V. parahaemolyticus* attackers at 30ºC on media containing 3% NaCl (MLB). Asterisks denote statistical significance between samples at the 4 h timepoint by an unpaired, two-tailed Student's *t*-test (* $P < 0.05$); RIMD, RIMD 2210633; DL, detection limit. Source data are provided as a source data file.

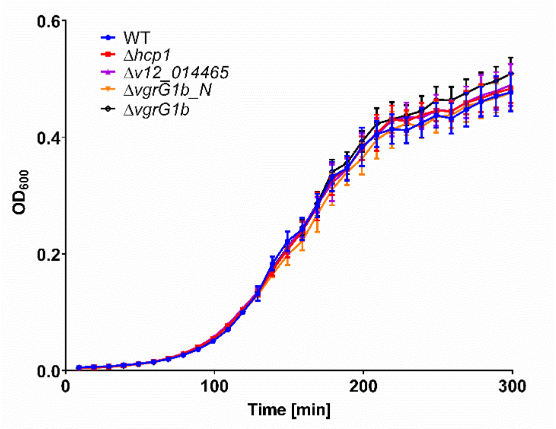

Supplementary Figure 3. *V. parahaemolyticus* 12-297/B gene deletions do not affect growth. Growth of *V. parahaemolyticus* 12-297/B deletions in MLB (LB with 3% NaCl) at 30ºC is shown as OD_{600} measurements. Data are mean ± S.D. (n=10). Δ*vgrG1b*_N, deletion of nucleotides encoding the first 724 amino acids of VgrG1b. Source data are provided as a source data file.

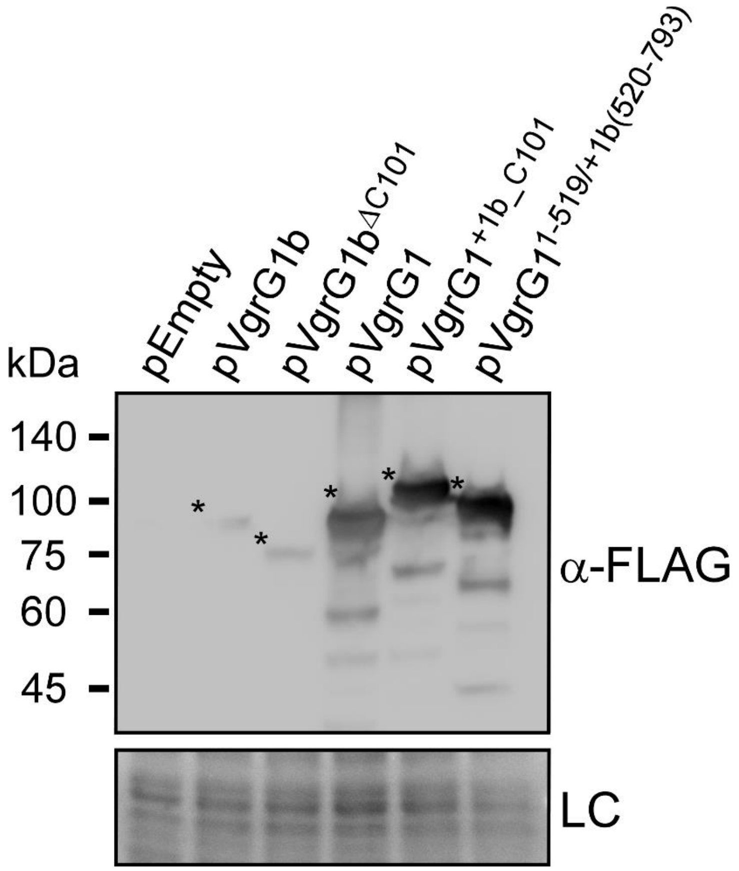

Supplementary Figure 4. VgrG forms are stably expressed ectopically in *V. parahaemolyticus* 12-297/B. Expression of indicated C-terminally FLAG-tagged forms of VgrG1 or VgrG1b in *V. parahaemolyticus* 12-297/B *ΔvgrG1b_N* mutant from arabinose inducible plasmid (with 0.01% L-arabinose). Strains were the same as used in the result shown in Fig. 1e. Proteins were detected by immunoblotting using specific antibodies against FLAG. Loading control (LC), visualized as trihalo compound's fluorescence of the immunoblot membrane, is shown for total protein lysates. Expected bands are denoted with an asterisk in the upper left corner. Source data are provided as a source data file.

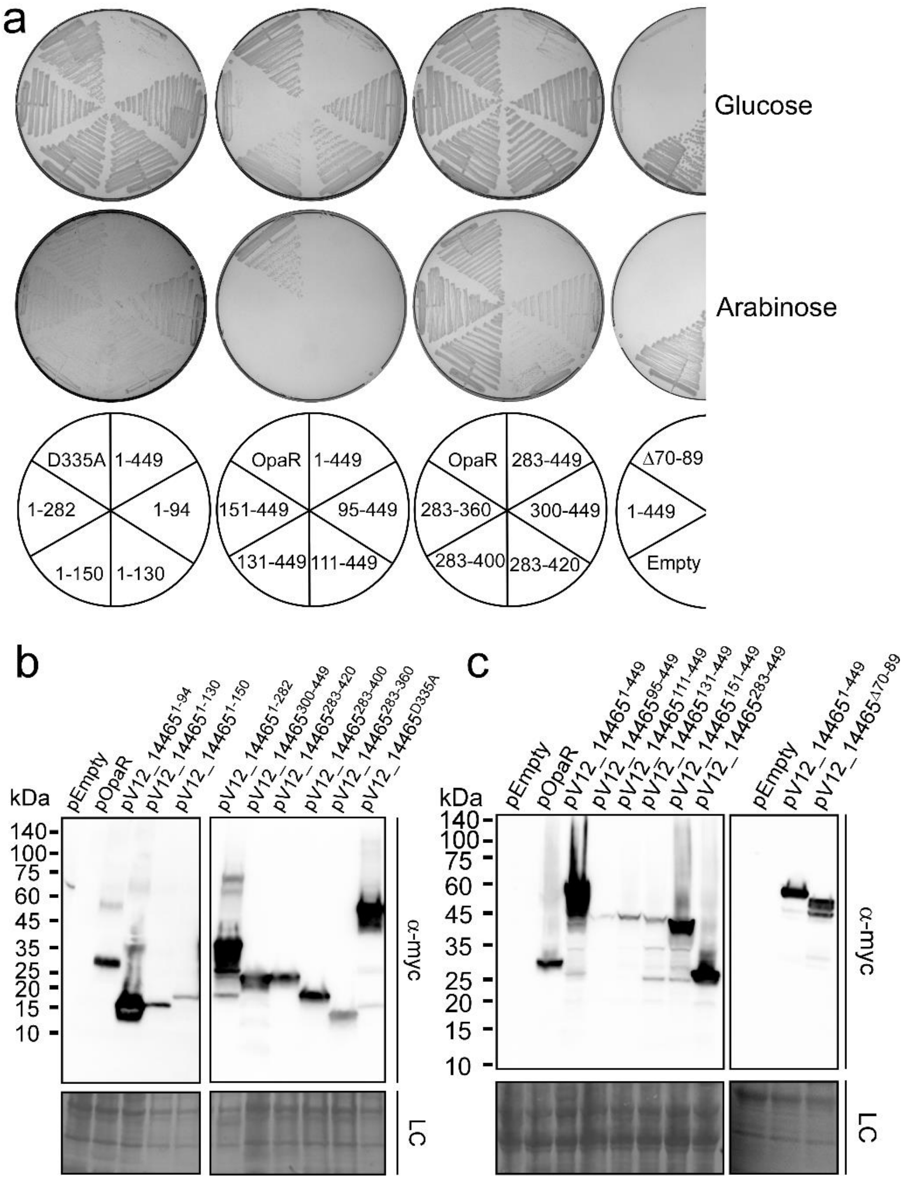

Supplementary Figure 5. The C-terminus of V12_14465 is required for toxicity in *E. coli*. a, Toxicity of V12_14465 variants expressed in *E. coli* BL21(DE3) from an arabinose-inducible expression plasmid. Amino acid ranges of expressed truncations are shown in the lower panel. OpaR, the *V. parahaemolyticus* high cell density quorum sensing master regulator, was used as a non-toxic control. **b-c,** Expression of indicated C-terminally myc-tagged forms of V12_14465 or of OpaR (as control) as in (a) expressed from arabinose inducible plasmid in *E. coli* BL21(DE3), without (**b**) or with (**c**) simultaneous expression of the immunity V12_14460 to allow detection of V12_14465 if deleterious to *E. coli* when expressed alone. Proteins were detected by immunoblotting using specific antibodies against myc. Loading control (LC), visualized by PonceauS staining of the immunoblot membrane, is shown for total protein lysates. Source data are provided as a source data file.

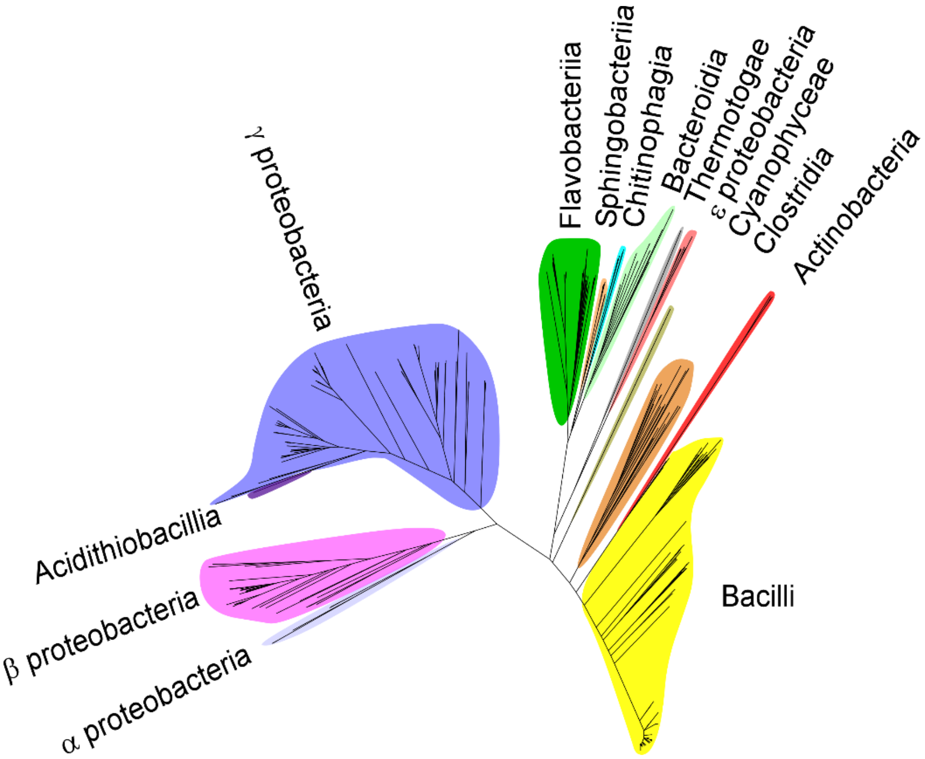

Supplementary Figure 6. PoNe domains are widespread in bacteria. Phylogenetic tree of bacteria encoding PoNe-containing toxins. The class of bacteria composing each colored branch is listed. The tree was based on the DNA sequence of *rpoB*.

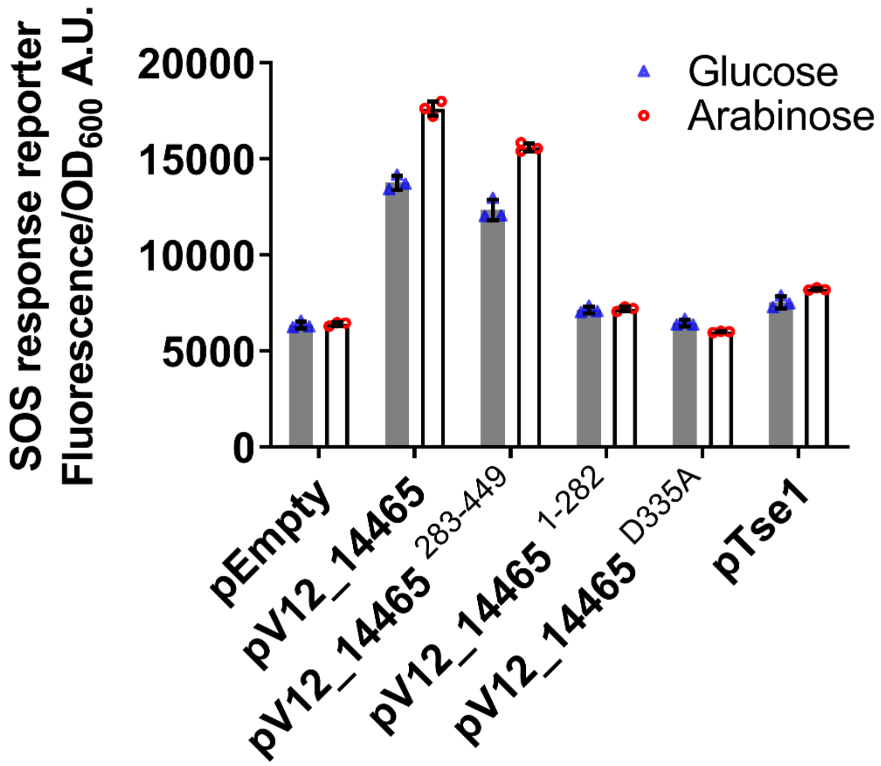

Supplementary Figure 7. PoNe domain of V12_14465 activates the SOS response in *E. coli*. SOS response activation in *E. coli* BL21(DE3) harboring the indicated arabinose-inducible plasmids. SOS response levels were determined as GFP fluorescence from the reporter plasmid pL(*lexO*)-GFP, divided by cell density (OD_{600}), and are shown as arbitrary units (A.U.). The peptidoglycan hydrolase effector Tse1 was used as a negative control. Data are mean ± S.D. (n=3). Source data are provided as a source data file.

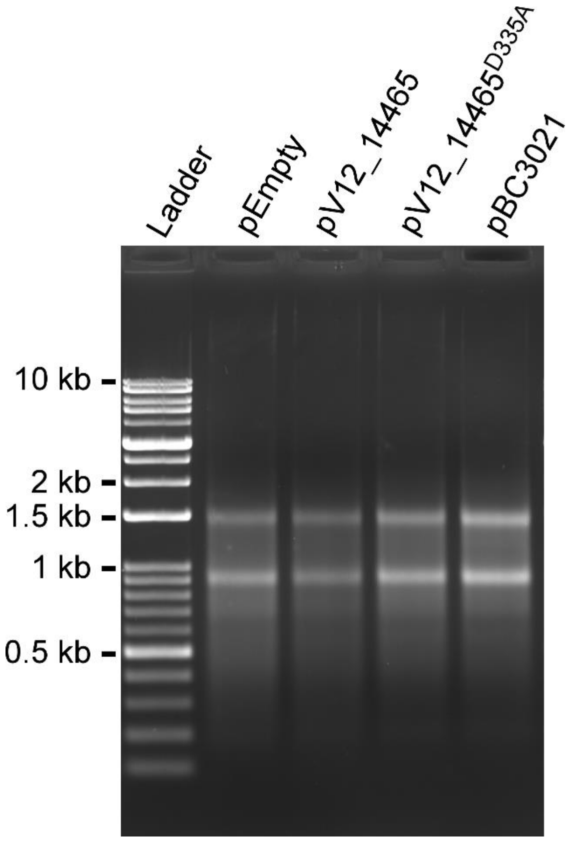

Supplementary Figure 8. PoNe does not target bacterial RNA. In vivo RNase activity assay. *E. coli* BL21(DE3) harboring expression plasmids for the indicated V12_14465 and BC3021 variants or an empty vector (pEmpty) were induced with L-arabinose for 1 h, and total RNA was purified. Integrity of RNA was visualized on 1% agarose gel.

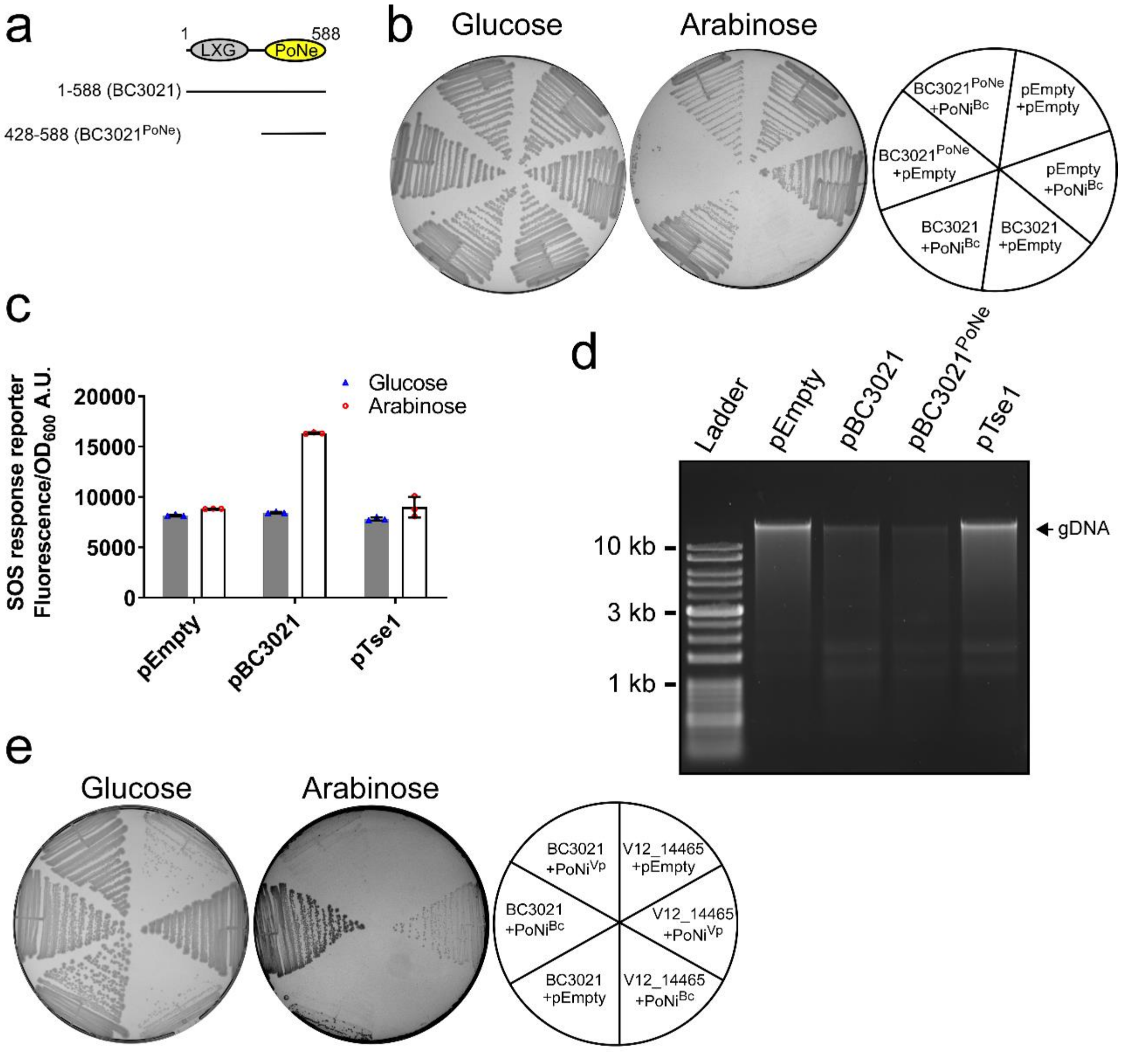

Supplementary Figure 9. *B. cereus* PoNe is a DNase that is antagonized by the immunity protein encoded downstream. a, Schematic representation of full-length and truncated PoNe-containing *B. cereus* BC3021. **b,** Toxicity of BC3021 variants expressed in *E. coli* BL21(DE3) from an arabinose inducible expression plasmid with or without BC3020 ($PoNi^{Bc}$). **c,** SOS response activation in *E. coli* BL21(DE3) harboring the indicated arabinose-inducible plasmids. SOS response levels were measured as GFP fluorescence from the reporter plasmid pL(*lexO*)-GFP divided by cell density (OD_{600}) and shown as arbitrary units (A.U.). The peptidoglycan hydrolase effector Tse1 was used as a negative control. Data are mean ± S.D. (n=3). **d,** In vivo DNase activity assay. *E. coli* BL21(DE3) harboring expression plasmids for the indicated BC3021 variants or Tse1 were induced with L-arabinose for 2 h, and genomic DNA (gDNA) was purified. Integrity of gDNA was visualized on 0.7% agarose gel. **e,** Toxicity of V12_14465 and BC3021 expressed in *E. coli* BL21(DE3) from arabinose inducible expression plasmid with or without V12_14460 ($PoNi^{Vp}$) or BC3020 ($PoNi^{Bc}$). Source data are provided as a source data file.
