# Supplementary material for: A modular effector with a DNase domain and a marker for T6SS substrates
Source: Nat Commun. 2019 Aug 9;10:3595. doi: 10.1038/s41467-019-11546-6 (PMC6688995; doi:10.1038/s41467-019-11546-6)
Supplement: Supplementary file 3 — Description of Additional Supplementary Files [file 41467_2019_11546_MOESM3_ESM.pdf]

Description of Additional Supplementary Files

File Name: Supplementary Data 1
Description: PoNe-containing toxins and adjacently encoded proteins identified in the analysis.

File Name: Supplementary Data 2
Description: FIX-containing proteins and adjacently encoded proteins identified in the analysis.

File Name: Supplementary Data 3
Description: Summary of T6SS core-components identified in genomes harboring FIX-containing proteins.
